# SRPX2 boosts pancreatic cancer chemoresistance by activating PI3K/AKT axis

**DOI:** 10.1515/med-2020-0157

**Published:** 2020-10-22

**Authors:** Zhenyuan Gao, Jisong Wu, Xiao Wu, Jialei Zheng, Yimei Ou

**Affiliations:** Department of Oncology, The First Affiliated Hospital of Bengbu Medical College, 287 Changhuai Road, Anhui, China

**Keywords:** SRPX2, PI3K/AKT/mTOR, pancreatic cancer, chemo-resistance, cell migration, cell invasion

## Abstract

**Background and aim:**

This investigation was aimed at disclosing whether SRPX2 affected pancreatic cancer (PC) chemoresistance by regulating PI3K/Akt/mTOR signaling.

**Methods:**

Totally 243 PC patients were recruited, and they were incorporated into partial remission (PR) group, stable disease (SD) group and progressive disease (PD) group in accordance with their chemotherapeutic response. PC cell lines (i.e. AsPC1, Capan2, VFPAC-1, HPAC, PANC-1, BxPC-3 and SW1990) and human pancreatic ductal epithelial cell lines (hTERT-HPNE) were also collected.

**Results:**

PC patients of SD + PD group were associated with higher post-chemotherapeutic SRPX2 level than PR group, and their post-chemotherapeutic SRPX2 level was above the pretherapeutic SRPX2 level (*P* < 0.05). PR population showed lower SRPX2 level after chemotherapy than before chemotherapy (*P* < 0.05). Besides high serum SRPX2 level and SRPX2 level change before and after chemotherapy were independent predictors of poor PC prognosis. Additionally, si-SRPX2 enhanced chemosensitivity of PC cell lines, and expressions of p-PI3K, p-AKT and p-mTOR were suppressed by si-SRPX2 (*P* < 0.05). IGF-1 treatment could changeover the impact of si-SRPX2 on proliferation, migration, invasion and chemoresistance of PC cells (*P* < 0.05).

**Conclusion:**

The SRPX2-PI3K/AKT/mTOR axis could play a role in modifying progression and chemoresistance of PC cells, which might help to improve PC prognosis.

## 1 Introduction

Pancreatic cancer (PC) was a gastrointestinal malignancy that appeared insidiously and yet deteriorated swiftly. Smoking, diabetes, tumor history, hepatitis B virus infection, over-consumption of nitrosamine and history of chronic pancreatitis were all its major risk factors [1]. In accordance with statistical estimation, PC was the fourth leading account for cancer-related deaths in America [2], and by 2030, it might become the second largest killer of cancer patients [3]. Although PC incidence was higher in developed countries than in developing countries [4], the adverse impact of PC on Chinese population could also not be underestimated [5]. One reason for this poor prognosis was that approximately 80–85% of PC patients have exacerbated to metastasis at the time of diagnosis, which made them unable to perform surgery [6]. Massive PC patients, therefore, were forced to seek for other treatments, and chemodrugs, such as gemcitabine and 5-fluorouracil (5-Fu), were among the choices [7,8]. Despite their outstanding role in treating advanced PC, drug-resistance that occurred during chemotherapy hindered remarkable improvement of PC prognosis [9,10]. Hence molecular mechanisms that explained PC chemoresistance demanded in-depth investigations.

Of note, SRPX2 was newly identified as a component of extracellular matrix (ECM) [11], which was a precisely ordered macromolecular network composed of cell secretory protein and polysaccharide and could efficaciously modify proliferation, adhesion, migration, differentiation and death of cells [12]. With these biological functions, ECM was implicated in neoplastic progression and infiltration [13], and curbing exchange between ECM and tumor cells was conducive to hindering tumor aggravation [14]. Accordingly, SRPX2 could also matter in regulating tumorigenesis, which has been verified in diversified tumors (e.g. PC) [15,16,17,18,19]. Nonetheless, whether and how SRPX2 played a part in modulating PC chemoresistance were still untapped.

In addition, PI3K/Akt signaling, which usually cooperated with mTOR to fuel tumorigenesis of PC [20,21], was implicated in the angiogenesis-inducing capability of SRPX2 [22]. Besides that, activation of this PI3K/Akt/mTOR signaling was usually followed by speedy growth and metastasis of tumors [23], and variation of its protein expressions was also indicative of tumor prognosis [24], which implied a linkage of SRPX2 with PI3K/Akt/mTOR signaling underlying tumorigenesis. Although PI3K/Akt/mTOR signaling has been corroborated to increase chemotolerance of cancer cells, such as PC and anaplastic thyroid carcinoma [25], documentations that elucidated the collaborative role of SRPX2 and PI3K/Akt/mTOR signaling in regulating PC chemosensitivity were scarce.

Therefore, this investigation was intended to disclose the interaction of SRPX2 and PI3K/Akt/mTOR signaling in modulating progression and chemoresistance of PC, which might provide a valuable alternative for PC treatment.

## 2 Materials and methods

### 2.1 Collection of PC samples

In the aggregate 298 PC patients, who sought medical advice in The First Affiliated Hospital of BengBu Medical College from February 2011 to January 2014 were incorporated. Their illness was classified based on the staging criteria laid down by the American Joint Committee on Cancer. All the PC patients satisfied the following inclusion criteria: (1) their clinical symptoms were consistent with items listed in the guidelines for diagnosis and treatment of PC (2014) [26]; (2) they were diagnosed as PC by tissue biopsy, exfoliative cytology and histopathology; and (3) their examination results of biochemical and blood routines were usual. Moreover, they would be ruled out if they were complicated by (1) primary disorders relevant to cardiovascular, liver, kidney and blood, (2) tumors located elsewhere, and (3) dysfunctions in mentality, coagulation system or hemorrhage. All the involvers have signed informed consents, and this program was approved by The First Affiliated Hospital of BengBu Medical College and its ethics committee.

### 2.2 Chemotherapy and efficacy evaluation of PC patients

The PC patients received first-line (i.e., gemcitabine) or second-line (i.e., FOLFOX4 regimen) drug treatments as recommended by the National Comprehensive Cancer Network. Efficacy of the chemotreatments was assessed in accordance with RECIST criterion. To be specific, complete remission symbolized that all target lesions were absent, and the level of tumor markers became normal. Partial remission (PR) was identified when long diameters of lesions were shortened by ≥30%, and progressive disease (PD) was decided if long diameters of lesions were increased by ≥30% or new lesions appeared. The PC patients would be judged as stable disease (SD) under the following conditions: (1) long diameters of lesions were decreased, yet PR could not be determined; (2) although long diameters of lesions were elongated, the extent was far from PD; and (3) several nontarget lesions were present, and/or tumor markers were above normal levels.

### 2.3 Determination of serum SRPX2 level in PC patients with ELISA kit

Around 5 mL of antecubital venous blood was collected from each PC patient before and after their chemotherapies. After standing at room temperature for 60 min, the blood samples were centrifuged at the speed of 900 g for 20 min. Then the serum level of SRPX2 was measured utilizing the ELISA kit manufactured by Wuhan Fine Biotech Corporation (Catalog number: EH12607, Hubei Province, China).

### 2.4 Cell culture

The PC cell lines, including AsPC1, Capan2, VFPAC-1, HPAC, PANC-1, BxPC-3 and SW1990, and human pancreatic ductal epithelial cell line (hTERT-HPNE) were all provided by American Type Culture Collection (ATCC). They were cultured in 5% CO_2_ at 37°C, and their culture medium was designated as DMEM/RPMI1640 medium (Gibco, USA) that included 10% fetal bovine serum (FBS, Gibco, USA).

### 2.5 Cell transfection and cell treatment

When BxPC-3 and SW1990 cell lines in the logarithmic phase were cultured to 80% confluence, 200 pmol si-SRPX2 (Genepharma, China) and 5 µL Lipofectamine 2000 reagent (Invitrogen, USA), respectively, diluted by 100 µL Opti-MEMI (Gibco, USA) were added to cultivate the PC cell lines for 48 h. As for insulin-like growth factor 1 (IGF-1) treatment, 40 nmol/L IGF-1 (Alexis Biochemical, Sweden), activator of PI3K/AKT/mTOR signaling was prepared to treat the BxPC-3 and SW1990 cell lines for 48 h.

### 2.6 MTT assay for assessing drug sensitivity of PC cells

BxPC-3 and SW1990 cell lines were inoculated into 96-well plates at a concentration of 1 × 10^4^ per well. After 24 h cultivation, the cells were treated by distinct concentrations of gemcitabine and 5-Fu for 48 h. Then 10 µL MTT solution (5 mg/mL, Invitrogen, USA) was poured to cultivate the cells for 4 h, after which cell supernatants were removed and the remaining sediments were dissolved by addition of dimethyl sulphoxide. Thirty minutes later, optical density values of each sample were gauged at the wavelength of 490 nm on a microplate reader. With drug concentration as the abscissa and cell survival as the ordinate, survival curves of the cells were drawn, and corresponding half-maximal inhibitory concentration (IC50) values [27] were also calculated.

### 2.7 CCK-8 assay

BxPC-3 and SW1990 cell lines of logarithmic growth were digested by trypsin and then centrifuged. Afterward the cells were adjusted to a concentration of 5 × 10^4^/mL by supplementation of 10% FBS-including culture medium. After cell culture for 24, 48 and 72 h, the PC cell lines were blended by 10% CCK-8 reagent (Takara, Japan). Four hours later, the PC cells were monitored on the microplate reader to obtain their absorbance (*A*) value at 450 nm.

### 2.8 Transwell invasion assay

The BxPC-3 and SW1990 cell lines at the concentration of 2 × 10^5^ per well were supplemented to the upper Transwell chamber (Corning-costar, USA) which contained polymerized Matrigel, while 400 µL FBS was supplemented to the lower section of Transwell chamber. After 48 h, cells that attached to the upper chamber were erased, whereas cells in the lower chamber were fixated by 95% ethanol and stained by 1% crystal violet for 10 min. The PC cells that went through the membrane were counted under the microscope, and the cell number in four views, which were randomly selected from the central and surrounding regions of the membrane, were averaged.

### 2.9 Wound healing assay

Until BxPC-3 and SW1990 cell lines were cultured to overspread the bottom of wells, the tip of a sterile microsyringe (model: 200 µL) was utilized to make a scratch. After 48 h of cell culture, distances of the scratches were measured.

### 2.10 Western blotting

The PC cell lines were dissociated on ice for 30 min after supplementation of lysis buffer. After centrifugation at the speed of 12,000 rpm at 4°C, cell supernatants were collected for quantitation of protein levels. Total proteins (30 µg for each sample) were separated after undergoing sodium dodecyl sulfate (SDS-PAGE; BIO-RAD, USA), and the resultant samples were then transferred onto the polyvinylidene fluoride membrane. With 5% skimmed milk applied to block protein samples for 2 h, primary antibodies (Abcam, USA) against β-actin (rabbit anti-human, 1:1,000, ab8226), PI3K (rabbit anti-human, 1:1,000, ab191606), p-PI3K (rabbit anti-human, 1:1,000, ab182651), Akt (rabbit anti-human, 1:10,000, ab179463), p-Akt (rabbit anti-human, 1:500, ab38449), mTOR (rabbit anti-human, 1:10,000, ab134903) and p-mTOR (rabbit anti-human, 1:1,000, ab109268) were diluted to incubate the proteins at 4°C for overnight. After that the samples were rinsed with TBST which was inclusive of Tween 20, and then secondary antibodies labeled by horseradish peroxidase (1:5,000, Abcam, USA) were utilized to incubate protein samples for 2 h. Then the samples were monitored with electro-chemiluminescence in the darkness, and gray values of protein bands were analyzed utilizing ImageJ software.

### 2.11 Statistical analyses

All the data were statistically analyzed adopting SPSS 17.0 software. Serum SRPX2 level, presented in the form of *M* (*P*_25_, *P*_75_), between groups was compared through Kruskal–Wallis test, and changes in serum SRPX2 level before and after chemotherapy were analyzed by Wilcoxon matched-pairs signed-rank test. Besides, Kaplan–Meier curves of PC patients were devised with log-rank test used for comparison, and clinical items that affected survival of PC patients were evaluated by uni/multivariate analyses. It was statistically significant in the case of *P* < 0.05.

## 3 Results

### 3.1 Association of SRPX2 with chemotherapeutic efficacy of PC patients

**Figure 1:**
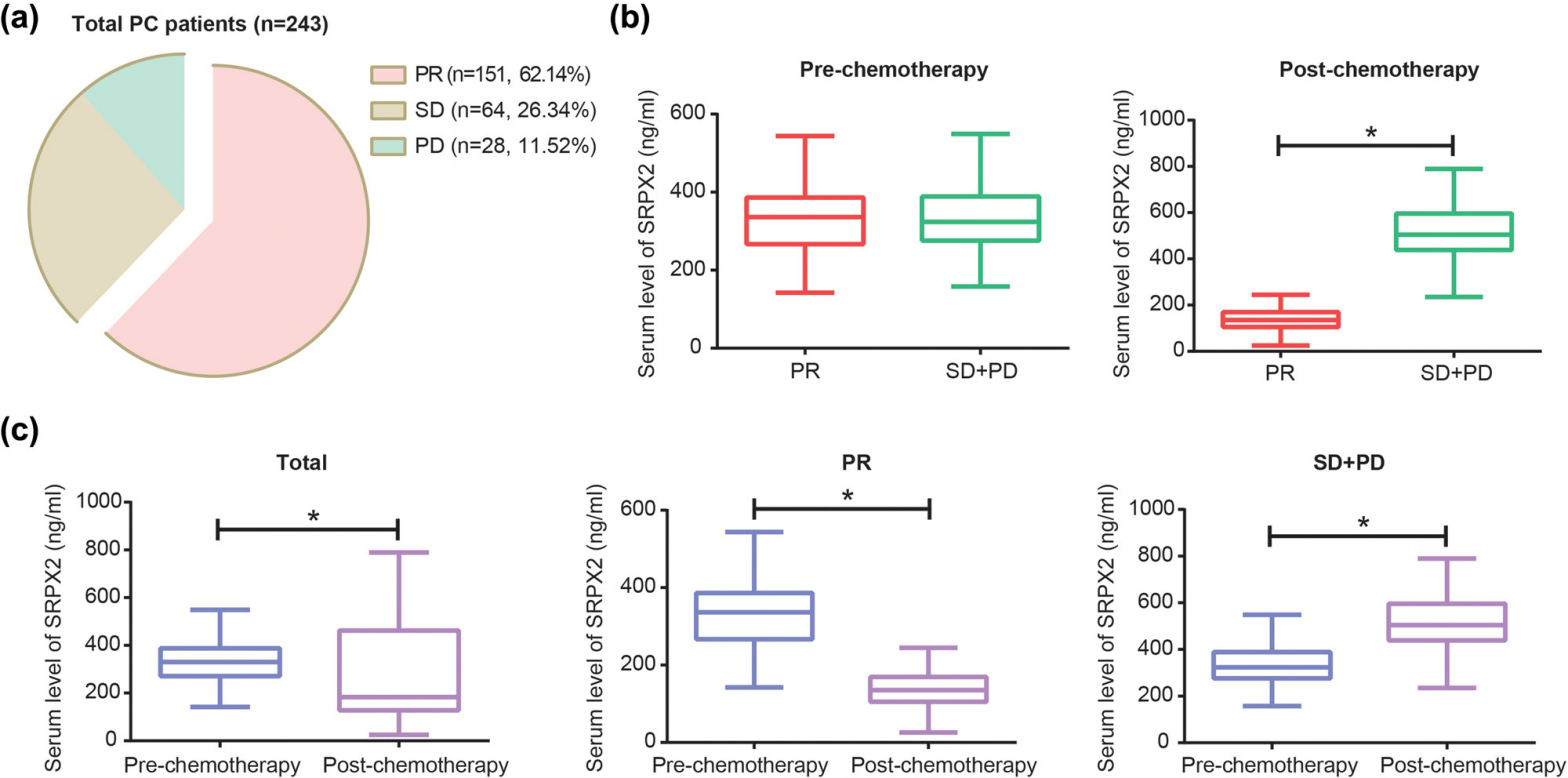
Correlation of SRPX2 with chemotherapeutic efficacy of PC patients. (a) The PC patients (*n* = 243) were tracked as partial remission (PR, *n* = 151), stable disease (SD, *n* = 64) and progressive disease (PD, *n* = 28). (b) Pre-chemotherapeutic SRPX2 level was compared between PC patients of PR group and ones of SD + PD group, and post-chemotherapeutic SRPX2 level was also contrasted between the two crowds. *P < 0.05. (c) Pre- and post-chemotherapeutic SRPX2 levels were compared, respectively, among total PC patients, ones of PR group and ones of SD + PD group. *P < 0.05.

After first/second-line drug treatments, 243 out of 298 PC patients were successfully followed up, with an effective follow-up rate of 81.54%. Of the 243 people, 151 patients were considered as PR, and the remaining ones were separately judged as SD (*n* = 64) and PD (*n* = 28) (Figure 1a). After chemotherapy, the SD + PD group [504.51 ng/mL (439.16, 595.30 ng/mL)] was associated with higher serum level of SRPX2 than PR group [135.41 ng/mL (105.46, 166.49 ng/mL)] (*z* = −13.064, *P* < 0.05), although no significant difference was observable regarding their pretherapeutic SRPX2 level (*z* = −0.225, *P* = 0.822) (Figure 1b). Besides, post-therapeutic SRPX2 level [182.95 ng/mL (128.18, 461.93 ng/mL)] of the whole population was markedly decreased as relative to their pretherapeutic SRPX2 level [330.25 ng/mL (272.65, 387.03 ng/mL)] (*z* = −3.841, *P* < 0.05) (Figure 1c). Similarly, SRPX2 level of SD + PD population after chemotherapy [504.51 ng/mL (439.16, 595.30 ng/mL)] was above that before chemotherapy [323.67 ng/mL (279.32, 388.27 ng/mL)] (*z* = −7.947, *P* < 0.05). Conversely, PR population was determined with lower SRPX2 level after chemotherapy [135.41 ng/mL (105.46, 166.49 ng/mL)] when compared with their prechemotherapeutic condition [336.14 ng/mL (268.97, 384.14 ng/mL)] (*z* = −10.646, *P* < 0.05).

### 3.2 Linkage of SRPX2 with prognosis of PC patients

**Figure 2:**
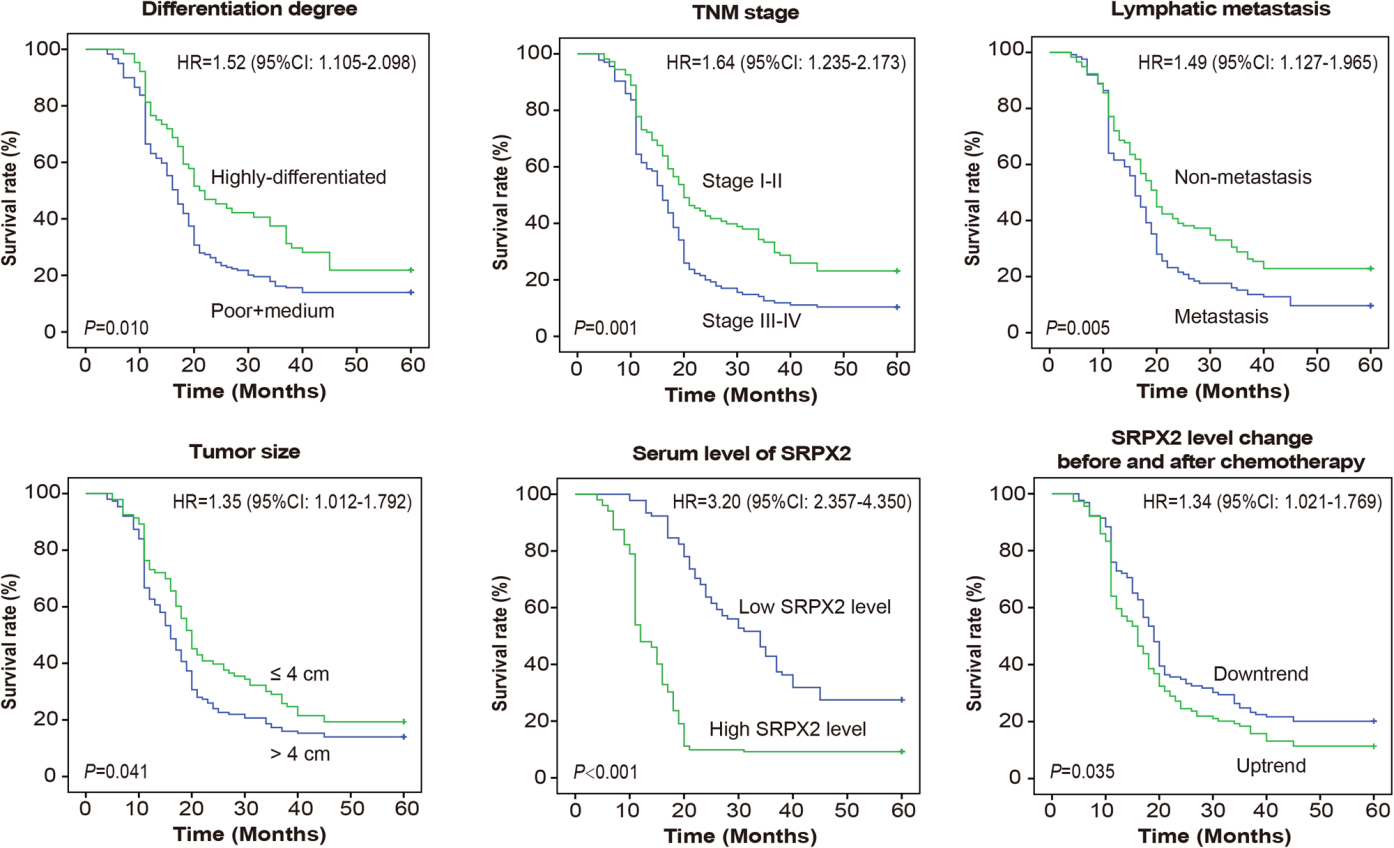
Prognosis of PC patients were compared when they were grouped according to differentiation level, TNM stage, lymphatic metastasis condition, tumor size, basal SRPX2 level and SRPX2 level change before and after chemotherapy. PC: pancreatic cancer.

As indicated in Kaplan–Meier curves (Figure 2), high differentiation, advanced TNM stage, lymphatic metastasis, large tumor size, high basal SRPX2 level and SRPX2 level change before and after chemotherapy were pronounced indicators of poor outcome among the recruited PC patients. The univariate Cox regression analyses also indicated that the six items were associated with 3 year survival of PC patients, while multivariate analysis presented that TNM stage, high serum SRPX2 level and SRPX2 level change before and after chemotherapy were independent involvers in forecasting unfavorable PC prognosis (all *P* < 0.05) (Table 1).

**Table 1:** Significance of clinico-pathological features in predicting 3-year survival of PC patients

Clinico-pathological features	Number of cases (*n*)	Univariate analyses			Multivariate analyses		
		HR	95% CI	*P* value	HR	95% CI	*P* value
**Gender (** ***n***)							
Male	110						
Female	133	1.181	0.897–1.556	0.237	1.175	0.889–1.553	0.258
**Age (years old)**							
<60	142						
≥60	101	0.98	0.741–1.296	0.889	1.023	0.77–1.357	0.877
**Tumor location (** ***n***)							
Ascending colon + transverse colon	180						
Descending colon + sigmoid colon	63	1.058	0.776–1.441	0.722	1.206	0.87–1.672	0.261
**Tumor size (cm)**							
≤4	93						
>4	150	1.347	1.012–1.792	0.041^a^	1.171	0.86–1.594	0.316
**Differentiation degree (** ***n***)							
High	64						
Moderate + poor	179	1.523	1.105–2.098	0.010^a^	1.204	0.863–1.68	0.276
**TNM stage (** ***n***)							
I + II	108						
III + IV	135	1.638	1.235–2.173	0.001^a^	1.56	1.157–2.103	0.004^a^
**Lymphatic metastasis (** ***n***)							
No	118						
Yes	124	1.488	1.127–1.965	0.005^a^	1.324	0.99–1.772	0.059
**Basal serum level of SRPX2 (** ***n***)							
Low-expressed	90						
High-expressed	153	3.202	2.357–4.35	<0.001^a^	3.045	2.195–4.225	<0.001^a^
**Change of serum SRPX2 level before and after chemotherapy**							
Downtrend	129						
Uptrend	114	1.344	1.021–1.769	0.035^a^	1.433	1.081–1.899	0.012^a^

HR: hazard ratio; CI: confidence interval.

^a^Statistical significance in the event of *P* < 0.05.

### 3.3 Silencing of SRPX2 sensitized PC cell lines against 5-Fu and gemcitabine by regulating PI3K/AKT/mTOR signaling

**Figure 3:**
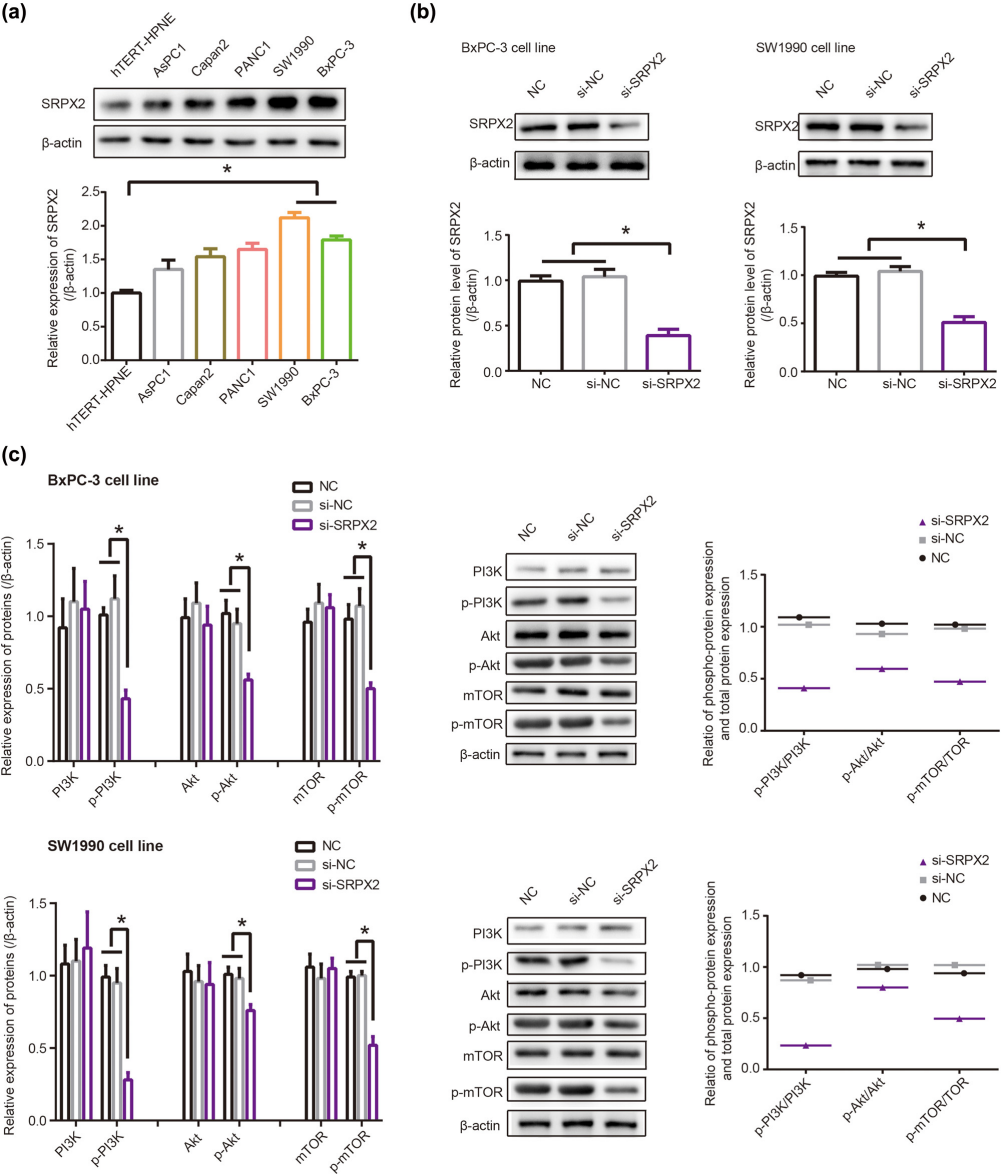
Influence of SRPX2 on PI3K/Akt/mTOR signaling. (a) Expression of SRPX2 was compared among hTERT-HPNE, AsPC1, Capan2, PANC1, SW1990 and BxPC-3 cell lines. *P < 0.05. (b) SRPX2 expression in SW1990 and BxPC-3 cell lines was determined after transfection of si-SRPX2. *P < 0.05. (c) Protein levels of PI3K, p-PI3K, Akt, p-Akt, mTOR and p-mTOR were monitored after transfecting si-SRPX2 into SW1990 and BxPC-3 cell lines. *P < 0.05.

SRPX2 expression went up significantly in PC cell lines (i.e., AsPC1, Capan2, PANC1, SW1990 and BxPC-3) in comparison with hTERT-HPNE cell line (*P* < 0.05), and it was maximized in SW1990 and BxPC-3 cell lines (*P* < 0.05) (Figure 3a). After transfection of si-SRPX2, expression of SRPX2 was dramatically lowered in BxPC-3 and SW1990 cell lines, when compared with that of NC group and si-NC group (*P* < 0.05) (Figure 3b). Moreover, silencing of SRPX2 decreased protein levels of p-PI3K, p-Akt and p-mTOR in BxPC-3 and SW1990 cell lines as relative to si-NC group (*P* < 0.05) (Figure 3c). On the contrary, p-PI3K, p-Akt and p-mTOR were over-expressed in BxPC-3 and SW1990 cell lines that were treated by IGF-1 (*P* < 0.05) (Figure S1). Besides that, ratios of p-PI3K/PI3K, p-Akt/Akt and p-mTOR/mTOR were decreased by si-SRPX2 (Figure 3c) and yet increased by IGF-1 (Figure S1), which suggested that PI3K/AKT/mTOR signaling was deactivated by si-SRPX2 and yet activated by IGF-1.

**Figure 4:**
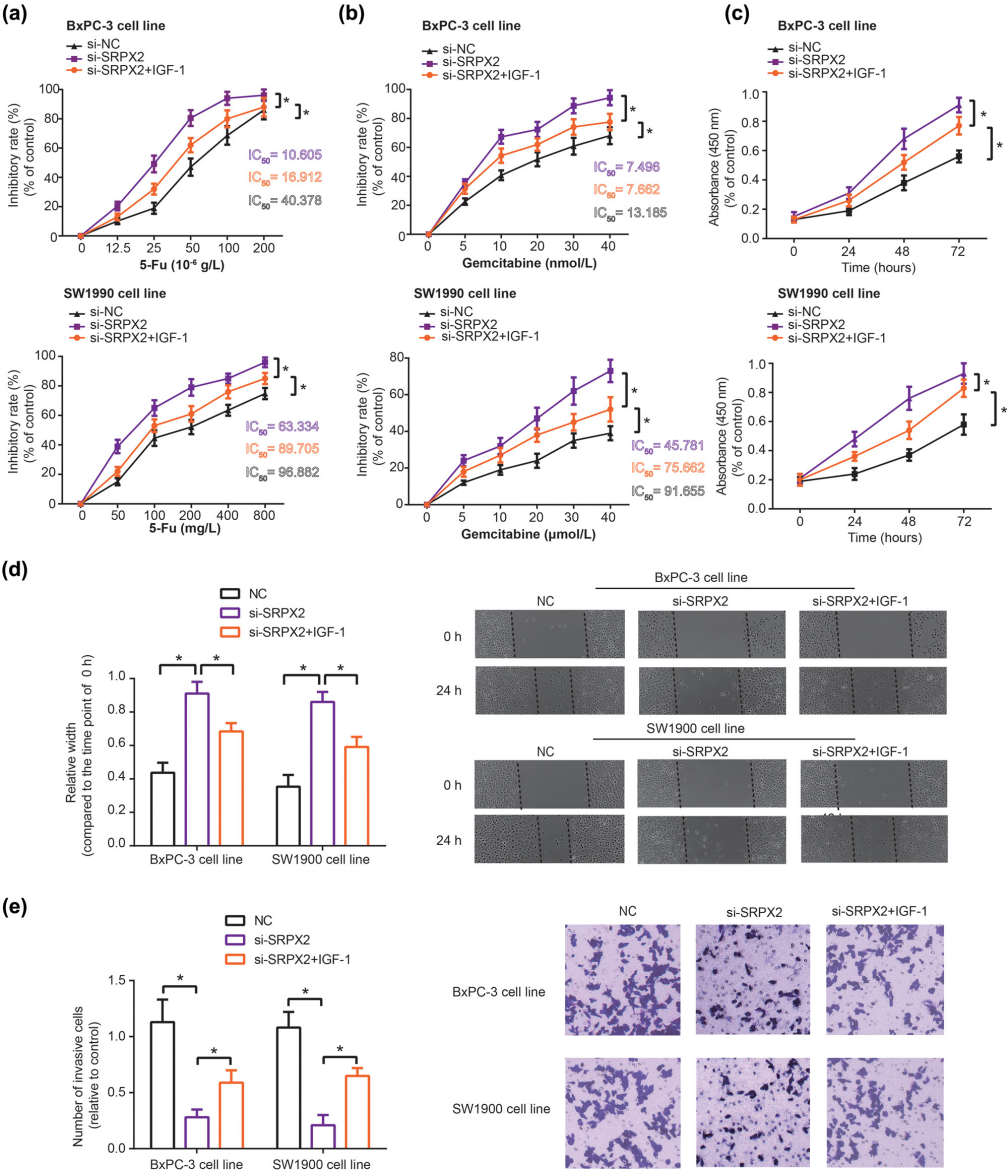
Chemo-resistance (a and b), proliferation (c), migration (d) and invasion (e) of SW1990 and BxPC-3 cell lines were evaluated among NC, si-SRPX2 and si-SRPX2 + IGF-1 groups. *P < 0.05.

Based on the above results, we further observed that 5-Fu-treated BxPC-3 and SW1990 cell lines in the si-SRPX2 group showed a decrease of proliferative power, as compared with si-NC group (*P* < 0.05) (Figure 4a). Migration and invasion of 5-Fu-treated BxPC-3 and SW1990 cell lines were also held back significantly by si-SRPX2 (*P* < 0.05) (Figure S2). Meanwhile, si-SRPX2 was found to alleviate tolerance of PC cell lines against gemcitabine, when compared with si-NC group (*P* < 0.05) (Figure 4b). However, BxPC-3 and SW1990 cell lines of si-SRPX2 + IGF-1 group were less vulnerable to the killing effect of gemcitabine and 5-Fu, as relative to BxPC-3 and SW1990 cells of si-SRPX2 (*P* < 0.05) (Figure 4a and b).

### 3.4 PI3K/AKT/mTOR signaling curbed the part of si-SRPX2 in regulating proliferation, migration and invasion of PC cells

When compared with NC group, proliferation of BxPC-3 and SW1990 cell lines was hindered by silencing of SRPX2 (*P* < 0.05) (Figure 4c). Results of wound healing assay also demonstrated that the migratory distance of CRC cell lines was widened in the si-SRPX2 group (*P* < 0.05) (Figure 4d), and the number of invasive PC cells also dropped markedly in the si-SRPX2 group in comparison to NC group (*P* < 0.05) (Figure 4e). Nonetheless, it was intriguing to observe that proliferation (Figure 4c), migration (Figure 4d) and invasion (Figure 4e) of BxPC-3 and SW1990 cell lines in the si-SRPX2 + IGF-1 group were intensified, when compared with cells of si-SRPX2 group (all *P* < 0.05).

## 4 Discussion

PC, a highly heterogenous cancer, has endangered massive population, and its 5 year overall survival was stuck at 5–8% despite medical advancements [28,29]. Surgical resection was a radical treatment for PC, yet this treatment could be available only for 15–20% of PC patients [4]. The remaining 70–80% PC patients, who already reached late stage as they were confirmed as PC, were no longer suitable to perform surgeries, and non-surgical treatments, such as chemotherapy, were required to prolong their survival [30]. However, chemotherapeutic failure, which was partly ascribed to chemotolerance of cancer cells, limited treatment effect of PC. Aggressive metastasis of cancer cells was an outstanding trigger of chemotherapeutic tolerance [31], which insinuated that molecular factors that halted tumor migration and invasion might alleviate chemoresistance.

A growing body of investigations has corroborated the participation of multifarious proteins in GEM-resistance. For instance, tolerance against GEM of cells was encouraged by p53 mutation [32], and activation of STAT5b boosted GEM-resistance by intensifying cell invasion [33]. Suppression of MRP2 expression also predisposed PC cells to be GEM-tolerant, for its activation of JNK, Erk1/2 and NF-kB signaling pathways [34]. Here SRPX2, a significant portion of ECM [14], was introduced, and it has been acknowledged to reinforce chemotolerance of tumors, such as temozolomide-resistance of glioblastoma and cisplatin-resistance of esophageal cancer [35,36]. In this study, we observed that high basal SRPX2 level and upward change of serum SRPX2 level were reflective of unfavorable prognosis among PC patients who received chemotherapies (Figures 1, 2 and Table 1), which implied that SRPX2 might be indicative of chemotherapeutic efficacy of PC patients. Furthermore, *in vitro* experiments demonstrated that the silencing of SRPX2 was responsible for decreased GEM/5-Fu-resistance of PC cell lines (Figure 4a and b), which might be explained by the role of si-SRPX2 in demotivating proliferation, migration and invasion of PC cells (Figure 4c and e). The metastasis-promoting role of SRPX2 was also manifested in colorectal cancer and liver cancer [37,38], which highlighted the oncogenic part of SRPX2. In summary, low serum SRPX2 level was corresponding to favorable prognosis of PC patients, possibly owing to its elevating PC chemo-sensitivity. However, this point entailed more proofs.

Another novelty of this investigation consisted in that PI3K/AKT/mTOR signaling was speculated as the downstream pathway of SRPX2 in intensifying migration, invasion and chemotolerance of PC cells (Figure 3). It was specifically embodied as that IGF-1, activator of PI3K/AKT/mTOR signaling, could, to some extent, reverse the effect of si-SRPX2 on proliferation, migration, invasion and gemcitabine/5-Fu-tolerance of PC cells (Figure 4). Virtually, the PI3K/AKT/mTOR signaling was crucial to modify tumor development and chemo-sensitivity. For instance, PI3K in collaboration with focal adhesion kinase (FAK) could powerfully spur cell metastasis, and PI3K inhibitor, on the contrary, disturbed the promotion of FAK on cell metastasis [39]. Moreover, PI3K signaling tended to increase expressions of matrix metal proteinases (MMPs), members of which, such as MMP-2 and MMP-9, were outstanding in degrading ECM and promoting migration and invasion of PC cells [40]. Not only that, the PI3K-led AKT/mTOR signaling also mattered in PC development by encouraging proliferation of PC cells [20].

Above all, this investigation introduced an SRPX2-PI3K/AKT/mTOR axis, which was implicated in modulating progression and chemo-resistance of PC, which might be helpful to develop PC-targeted treatments. Nevertheless, several defections should be improved in future. First, the size of included PC patients was limited, and all the PC population were of Chinese ethnicity, which made the study results less convincing and hard to be generalized to other populations. Second, apart from 5-Fu (Figure S2), whether SRPX2 combined with other chemodrugs exerted sizeable effects on PC metastasis was also worthy of exploration. Third, *in vivo* experiments were not conducted to verify the contribution of SRPX2-PI3K/AKT/mTOR axis underlying PC etiology. These points should be perfected to enhance the credibility of this investigation.
